# Automatic prediction of stroke treatment outcomes: latest advances and perspectives

**DOI:** 10.1007/s13534-025-00462-y

**Published:** 2025-02-17

**Authors:** Zeynel A. Samak, Philip Clatworthy, Majid Mirmehdi

**Affiliations:** 1 Department of Computer Engineering, Adiyaman University, 02040 Adiyaman, Turkey; 2 School of Computer Science, University of Bristol, Bristol, BS8 1UB UK; 3 Translational Health Sciences, University of Bristol, Bristol, BS8 1UD UK; 4 Stroke Neurology, Southmead Hospital, North Bristol NHS Trust, Street, Bristol, BS8 1UD UK

**Keywords:** Stroke, Outcome prediction, Treatment outcome, Functional outcome, Final infarct, Deep learning, Medical image analysis

## Abstract

Stroke is a major global health problem that causes mortality and morbidity. Predicting the outcomes of stroke intervention can facilitate clinical decision-making and improve patient care. Engaging and developing deep learning techniques can help to analyse large and diverse medical data, including brain scans, medical reports, and other sensor information, such as EEG, ECG, EMG, and so on. Despite the common data standardisation challenge within the medical image analysis domain, the future of deep learning in stroke outcome prediction lies in using multimodal information, including final infarct data, to achieve better prediction of long-term functional outcomes. This article provides a broad review of recent advances and applications of deep learning in the prediction of stroke outcomes, including (i) the data and models used, (ii) the prediction tasks and measures of success, (iii) the current challenges and limitations, and (iv) future directions and potential benefits. This comprehensive review aims to provide researchers, clinicians, and policy makers with an up-to-date understanding of this rapidly evolving and promising field.

## Introduction

Stroke is a serious medical condition that arises when the blood supply to the brain is disrupted. This interruption prevents oxygen from reaching brain cells, resulting in tissue damage, impaired neurological function, and potentially cell death. As a result, strokes can cause permanent brain damage, long-term disability, or even death. Several factors increase the likelihood of a stroke, including high blood pressure, diabetes, high cholesterol, smoking, heart disease, obesity and a family history of stroke. Age, gender, and ethnicity also contribute to the risk [1].

Stroke is the second leading cause of death and the third leading cause of disability worldwide, affecting approximately 15 million people annually [2], according to the World Health Organization [3]. The well-known phrase "time is brain" [4] highlights the critical importance of acting quickly in the assessment and treatment of stroke. Delays in treatment can lead to brain cell death from lack of blood flow and oxygen, and research suggests that approximately two million neurons die every minute an ischaemic stroke goes untreated [4]. A comprehensive analysis of registry data revealed that each minute delay in intravenous thrombolysis administration increases the risk of early post-treatment intracerebral haemorrhage, disability, and mortality [5]. Clinicians therefore aim to rapidly use all available data sources, including imaging and clinical information, to determine the most appropriate treatment option as quickly as possible.

The outcome for stroke patients is influenced by a number of factors, including the type, location and size of the stroke, the time elapsed before treatment and the rehabilitation interventions received [6]. Despite significant advances in understanding the mechanisms of stroke and developing effective treatments, like thrombectomy (mechanically removing blood clots, Endovascular Therapy (EVT)) and thrombolysis (dissolving blood clots with medication), predicting patient outcomes and selecting the most appropriate treatment remains challenging because it involves complex interactions between multiple variables that may change over time [7]. Consequently, developing an automated tool that uses available imaging and patient information to predict treatment outcomes would improve clinical decision-making and the efficacy of ischaemic stroke treatment.

**Fig. 1:**
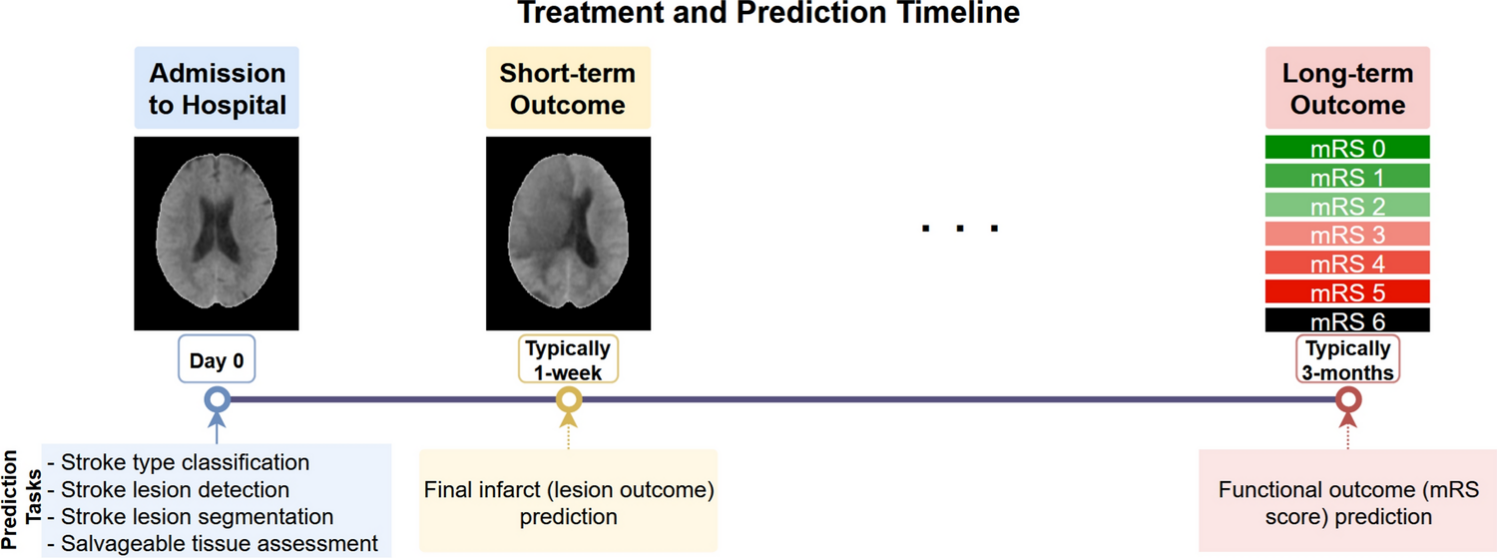
Analysis of stroke time frames and the predicted tasks at each time point. These predictions are based on initial scans performed upon hospital admission to aid physicians in their clinical decision-making process

The early prediction of stroke outcome and delivery of the appropriate treatment is critical since the ischaemic penumbra, the region of brain tissue surrounding the infarct that can still be saved, has a limited window for successful intervention [8]. To address this, researchers categorise stroke analysis into distinct phases and tasks (see also Fig. 1) to guide treatment decisions [9, 10]. The initial assessment occurs upon hospital admission to identify brain tissue damage. Subsequent analyses are conducted to evaluate how the tissue is changing in response to treatment and determine longer-term functional outcomes, typically within the first week and at three months. Importantly, all predictions within these time-frames must be made after the initial hospital admission scan to help clinicians choose the most appropriate course of treatment.

To make accurate predictions within these time-frames, researchers use a variety of data sources: brain imaging techniques such as Computed Tomography (CT) scans (used for rapid stroke assessment), Magnetic Resonance Imaging (MRI) (providing detailed anatomical information) and clinical data including patient demographics, blood biomarkers and neurological scores. Given the requirement of quick reactions to determine diagnosis and perform corrective procedures, clinical and radiological *reports* are either not available or feature less in such situations. Hence, there is also a lack of such data for researchers to apply today’s enhanced language models [11, 12] for outcome predictions. However, this is an important area which has scope for development.

Early attempts to automate stroke analysis relied on basic techniques such as thresholding and standard statistical methods. These methods included correlation-based analysis [13, 14], region growing [14, 15], and classical Machine Learning (ML) algorithms such as Random Forest (RF) [16, 17] and Support Vector Machine (SVM) [18]. In recent years, ML and Deep Learning (DL) methods have gained significant popularity due to their effectiveness in various image analysis tasks, such as object detection [19, 20], image segmentation [21, 22], and classification [23, 24]. DL, in particular via Convolutional Neural Network (CNN) [25–27] and recently Transformers [28–30], has been widely applied to medical image analysis tasks with superior results, including automated ischaemic stroke lesion classification [31, 32], detection/segmentation [25, 27] and prognosis [33–35]. Most Stroke Outcome Prediction (SOP) studies have focused on either predicting the final lesions or predicting functional outcome at 90-days. While [36] demonstrated the 1-week follow-up scan containing final lesions has a positive impact on predicting the functional outcome, few studies have investigated integrating the final lesion and functional outcome prediction tasks [35, 37].

**Table 1:** Recent review/survey articles that included Stroke Outcome Prediction (SOP), their coverage, topics reviewed, total number of papers reviewed, including total number of articles related to SOP

Study	Year	Coverage	Reviewed topics	#Papers	#DL in SOP
[38]	2020	2007–2019	ML in stroke identification, diagnosis, treatment & prognostication	39	1
[8]	2020	To 2020	Stroke lesion detection and segmentation	113	7
[9]	2021	2010–2021	Detection and lesion segmentation of neuroimage	117	7
[39]	2021	To 2021	ML in stroke diagnosis and outcome prediction	54	3
[10]	2022	To early 2022	Functional outcome, reperfusion, and hemorrhagic transformation	16	4
[40]	2022	To mid-2020	Functional Outcome after EVT and using MR CLEAN dataset	19	2
[41]	2022	To mid-2022	ML algorithms for final infarct prediction	11	7
[42]	2022	To mid-2022	AI applied in stroke decision support	65	11
Our review	**2024**	**2015–2024**	**DL in SOP (final infarct&functional outcome)**	**60**	**60**

Several previous review papers (summarised in Table 1) have investigated the application of ML and DL techniques in the domain of brain stroke. While these prior reviews have comprehensively addressed stroke classification [38, 39], detection [8, 9], and segmentation [8, 42] and many have focused on classical ML methods [38, 39, 41], the application and progress of DL in SOP has not been coalesced and categorised.

In this paper, we present an overview of the different DL approaches that have been applied to predict the outcome of stroke treatment as the final lesion and functional outcome using only imaging and multimodal data. We discuss the strengths and limitations of these techniques and critically evaluate the current state of the field, identifying gaps and areas that require further investigation to improve the precision and reliability of SOP. Additionally, we consider the public datasets that are available for SOP applications, as well as list the works for which code (see Appendix Table 7) and data are available.

The rest of the paper is structured as follows. In Sect. 2, we consider existing, relevant datasets and score measures for stroke outcome prediction. Section 3 reviews articles in SOP using DL methods under two categories: final infarct and functional outcome prediction. In the Discussion in Sect. 4, we present our key observations and review current limitations. Then, in Sect. 5, we suggest avenues of further exploration for future directions of research for more efficient stroke outcome prediction. Finally, Sect. 6 concludes the paper.

## Datasets & scores

Publicly available datasets are scarce within the SOP domain, hence some studies use their own in-house collections. Table 2 lists only datasets that have been used in at least two studies of SOP research. While access to most of these datasets requires application and approval, the Ischemic Stroke Lesion Segmentation (ISLES) 2017 [43] dataset, released for a challenge at the MICCAI^1^ conference, stands out as a publicly available resource, providing valuable data for developing SOP models and benchmarking for predicting stroke lesion outcome using MRI data.

**Table 2:** Chronological overview of datasets used for SOP

Study	Year	Availability	Image modality	Patients	Final Infarct	Functional outcome	Articles used
I-KNOW [44, 45]	2009	N/A	MRI	168	✓	✓	4
DEFUSE-2 [46]	2012	On Request	MRI	104	✓	✓	3
HERMES [47]	2016	On Request	NCCT,CTA	1287	✗	✓	2
ISLES 2017 [43]	2017	Public	MRI Sequences	75	✓	✗	6
CRISP [48]	2017	N/A	CTP	190	✓	✓	3
iCAS [49]	2019	On Request	CBF	77	✗	✓	3
ERASER [50]	2019	On Request	NCCT,CTP	80	✓	✓	4
MR CLEAN							
Trial [51]	2015	On Request	NCCT,CTA	500	✗	✓	5
Registry [52]	2018	Private	NCCT,CTA	1488*	✗	✓	2
NO IV [53]	2021	On Request	NCCT,CTA, DWI	539	✗	✓	1
HIBISCUS-STROKE [54]	Ongoing	On Request	MRI, CT	164	✓	✓	2

"On request" indicates that access to associated dataset is subject to a formal application process. N/A means that corresponding information was not available. * First cohort of the registry

The ISLES 2017 [43] dataset comprises MRI diffusion maps (Diffusion-weighted MRI (DWI), Apparent Diffusion Coefficient (ADC)) and perfusion maps (Cerebral Blood Volume (CBV), Cerebral Blood Flow (CBF), Mean Transit Time (MTT), Time To Peak (TTP), Time-to-Maximum (Tmax)) as well as clinical details, such as time since stroke onset, time to treatment, Thrombolysis in Cerebral Infarction (TICI) score, and modified Rankin Scale (mRS) score. Additionally, ground truth labels include segmentation maps acquired from follow-up stroke imaging are also available. The training dataset consists of 43 patients, with results assessed on a separate test set of 32 stroke patients.

The Highly Effective Reperfusion Evaluated in Multiple Endovascular Stroke Trials (HERMES) [47] collaboration was established to combine patient-level data from five key trials (MR CLEAN [51], ESCAPE [55], REVASCAT [56], SWIFT PRIME [57], and EXTEND IA [58]) totalling 1287 patients, admitted between late 2010 and late 2014. The trial investigators aimed to answer outstanding questions about the effectiveness of thrombectomy in different groups of patients with large-vessel ischaemic stroke. The primary outcome of the trial was functional outcome at 90 days.

The Multicenter Randomized Clinical trial of Endovascular treatment for Acute ischemic stroke in the Netherlands (MR CLEAN) [51] dataset was collected as part of a randomised clinical trial that compared intra-arterial treatment to usual care for patients with proximal arterial occlusion in the anterior circulation, and includes data from 500 patients treated at 16 medical centres in the Netherlands. The dataset includes a baseline (*i.e. * the first scan when the patient was admitted to the hospital) Non-contrast Computed Tomography (NCCT) and CT Angiography (CTA), 24-hour follow-up NCCT and CTA scans, and additionally a 1-week follow-up NCCT scan. Additionally, MR CLEAN contains clinical metadata information such as patient demographics (age, gender), medical history and stroke scores (*e.g. * National Institutes of Health Stroke Scale (NIHSS)), imaging biomarkers (*e.g. * Alberta Stroke Programme Early CT Score (ASPECTS)) and outcome data mRS.

### mRS score

The mRS score is used to assess the success rate and functional outcome of stroke treatment [59, 60] and is based on the degree of disability at 90 days after a patient received stroke treatment. It is assessed by an independent observer and scored on a standardised scale as seen in Table 3. Initially, mRS was a 5-point scale ranging from 1 to 5 [59], later becoming a 6-level scale by adding 0 for patients with no symptoms [60], and recently mRS has become a 7-point scale that varies from 0 (no symptoms) to 6 (death of patient) [61]. A mRS score between 0-2 indicates a good outcome, characterised by functional independence, which means that a patient is able to perform daily activities without requiring assistance from another person. This also means they were favourable to treatment and that the treatment was successful. On the other hand, a mRS score between 3 and 6 represents a bad outcome and functional dependence.

**Table 3:** The modified Rankin Score - a basic explanation of each mRS score

Score	Grade of disability
0	No symptoms at all
1	No significant disability despite symptoms; able to carry out all usual duties and activities
2	Slight disability; unable to carry out all previous activities, but able to look after own affairs
3	Moderate disability; requires some help, but able to walk without assistance
4	Moderately severe disability; unable to walk and to attend to own bodily needs without assistance
5	Severe disability; bedridden, incontinent and requiring constant nursing care and attention
6	Dead

### National institutes of health stroke scale (NIHSS)

The NIHSS is a standardised tool used by healthcare professionals to assess the severity of stroke by way of a 15-item examination that assesses a patient’s level of consciousness, speech, motor skills, vision, balance and sensory function. Each item is scored between 0 (normal) and a maximum depending on the specific function (e.g. 4 for worst motor strength), with higher total scores (0–42) indicating greater stroke severity. This score is used to guide treatment decisions, such as clot-busting medication or clot removal surgery, and to monitor a patient’s recovery. Importantly, the effectiveness of the NIHSS may be limited by the patient’s level of cooperation. As the scale relies on the patient’s ability to follow instructions and communicate, those with stroke-related impairments or pre-existing neurological conditions may not be accurately assessed on arrival at hospital or during subsequent assessments.

### Thrombolysis in cerebral infarction (TICI)

The TICI scale provides a standardised method for grading blood vessel re-opening using digital subtraction angiography (DSA) imaging in interventional radiology following stroke treatment, particularly thrombectomy. This scale, ranging from 0 (no flow) to 3 (complete reperfusion), allows doctors to assess the success of clot removal based on the observed blood flow patterns. Higher scores ( 2b and 3) indicate a successful procedure and a greater likelihood of positive patient outcomes.

### Alberta stroke programme early CT score (ASPECTS)

The ASPECTS score is a widely used tool for assessing the extent of early ischaemic changes in patients with Acute Ischaemic Stroke (AIS). This score is derived from NCCT scans and quantifies the volume of brain tissue affected by ischaemia. The score is determined by the presence or absence of ischaemic changes in specific brain regions, scales from 0 to 10. Higher score correlates with better functional outcomes, making it useful information for predicting immediate treatment response and short-term prognosis [62–64]. However, it is important to note that the primary purpose of ASPECTS is to evaluate initial brain damage and inform immediate treatment decisions, rather than evaluating long-term treatment effectiveness like mRS [65, 66].

## Deep learning for stroke outcome prediction

In recent decades, a variety of techniques, from image thresholding [13] to DL [25, 31, 32], have been utilised for stroke lesion detection, segmentation, and classification. However, the focus has shifted now beyond segmenting lesions to using all available information to specifically predict stroke treatment outcomes [26, 34, 35].

Previous research on predicting treatment outcomes using patient imaging and/or clinical data concentrates on two main areas: (1) the evolution and final appearance of stroke lesion volume and (2) the prediction of mRS scores. This section reviews and summarises key contributions, methods, and applications from previous studies, categorised by their *prediction target* (lesion and functional outcome) and *data type* (imaging only and imaging with clinical information) used for estimating stroke treatment outcomes. Thus next, Sect. 3.1 addresses studies that assess stroke treatment success by predicting the final lesion and Sect. 3.2 then reviews studies that predict functional outcomes, mRS scores after stroke treatment.

### Final infarct prediction

This section focuses on predicting stroke treatment outcomes by estimating the final appearance of the stroke lesion on follow-up scans. While follow-up scans can be acquired within a broad time frame ranging from 1 h to 90 days [41, 71, 91], most studies generally adopt a time point of one week (5–7 days) [76, 86, 87, 92, 93]. Researchers have mainly addressed this task by developing models trained on datasets of expert-annotated follow-up scans. These models analyse a patient’s initial scan and predict the final lesion segmentation map, which is essentially a visual representation of the extent of the stroke. This information provides physicians with a valuable tool to develop more effective treatment plans. Throughout this article, the terms "final infarct", "tissue fate", "follow-up lesion" and "final lesion" are used interchangeably to mean the same outcome and to remain consistent with terms used in the community.

**Table 4:** Overview of the studies that perform final infarct prediction as stroke treatment outcome.

Study	Year	Patient subgroup	Treatment type	Method	Input modality	Number of patients	Data split	Best result
[67]	2015	AIS	EVT	CNN	MRI, Tmax	19	LOOCV	AUC: 0.86
[68]	2016	AIS	N/R	U-Net	MRI	49	19 test	AUC: 0.74
					Sequences		cases	DSC: 0.57
[69]	2018	AIS	rtPA	CNN	MRI	222	85:15	AUC: 0.88
[70]*	2018	AIS	EVT	U-Net	CTP	29	5-fold	DSC: 0.46
				+ CAE			cv	
[71]	2018	AIS	EVT	multi U-Net	DWI	75	32 test	DSC: 0.29
				+ GRU	PWI		cases	
[72]	2019	MCA	EVT	CNN	MRI, PWI	48	10-fold	AUC: 0.871
					FLAIR		cv	
[73]	2019	Anterior	tPA	CNN	Synthetic	100	8 test	DSC: 0.45
		circulation			PWI	simulations	cases	
[74]	2019	AIS	EVT	CNN	CT	1026	396 test	ICC: 0.88
							cases	DSC: 0.57
[75]	2020	Anterior	tPA	CNN	PWI	125000	8 test	DSC: 0.40
		circulation				patches	cases	
[76]	2020	Anterior	EVT	CNN	MRI	182	5-fold	AUC: 0.92
		circulation			Sequences		cv	DSC: 0.53
[77]	2020	AIS	EVT	CNN	CTP	29	5-fold	F1: 0.74
				+ PCA			cv	
[78]	2020	LVO	EVT	CNN	CTP	188	5-fold	DSC: 0.48
				DeepMedic			cv	
[79]	2021	AIS	EVT	End2End	DSC-MRI	75	32 test	DSC: 0.31
				CNN			cases	
[54]	2021	LVO	EVT	Multi-scale	DWI	109	5-fold	AUC: 0.87
				U-Net	PWI		cv	DSC:0.44
[80]	2021	LVO	EVT	CNN	CTA	89	50 test	DSC: 0.60
							cases	
[27]*	2021	AIS	EVT	RBM	MRI	75	32 test	DSC: 0.38
				CNN	Sequences		cases	
[81]	2021	AIS,	tPA	Ensemble	DWI	40	70:30	AUC: 0.898
				U-Net + ML				
[29]	2022	AIS	EVT	U-Net	4D CTP	147	10-fold	DSC: 0.45
				Temporal-CNN			cv	
[82]	2022	LVO	EVT	U-Net	CTP	110	60:20:20	AUC: 0.93
								DSC:0.67
[83]	2022	LVO	EVT	U-Net	CTP	145	68:12:20	DSC: 0.289
			tPA					
[84]*	2022	AIS	EVT	CNN +	CTP	45	10-fold	DSC: 0.45
				Transformer			cv	
[85]	2022	LVO	EVT	CNN	CTP	127	5-fold cv,	AUC: 0.88
				DeepMedic			101 ext. val	
[86]*	2022	AIS	tPA	U-Net	DWI	472	5-fold cv	DSC: 0.486
					PWI		55 ext. test	
Nazari *et al. * [87]	2023	AIS	EVT	Attention-gate	DWI	445	5-fold	AUC: 0.91
			tPA	U-Net	ADC		cv	DSC:0.50
[88]	2024	AIS	EVT	CNN	CTP	70	10-fold cv	DSC: 0.26
				CrossAttention				
[89]	2024	AIS	EVT	U-Net	NCCT	404	80:20	DSC: 0.37
					CTA		5-fold cv	
[90]	2024	AIS	EVT	CGAN	NCCT	147	5-fold cv	DSC: 0.37
			tPA		CTP			

Ischaemic Stroke (IS), Acute Ischaemic Stroke (AIS), Middle Cerebral Artery (MCA), Large Vessel Occlusion (LVO), Endovascular Therapy (EVT), recombinant tissue-type Plasminogen Activator (rtPA), tissue Plasminogen Activator (tPA), Convolutional Neural Network (CNN), Convolutional Auto Encoder (CAE), Conditional Generative Adversarial Network (CGAN), Principal Component Analysis (PCA), Restricted Boltzmann Machines (RBM), Magnetic Resonance Imaging (MRI), Time-to-Maximum (Tmax), CT Perfusion (CTP), Diffusion-weighted MRI (DWI), Perfusion-weighted Imaging (PWI), Fluid-attenuated Inversion Recover (FLAIR), Computed Tomography (CT), Noncontrast Computed Tomography (NCCT), CT Angiography (CTA), Apparent Diffusion Coefficient (ADC), Leave-One-Out Cross-Validation (LOOCV), Cross-Validation (cv), Area Under the Curve (AUC), Dice Similarity Coefficient (DSC), Intraclass Correlation Coefficient (ICC), Not Reported (N/R). * indicates that the code used in the study is available

An overview of various studies examined for final infarct prediction is presented in Table 4. Advanced imaging techniques, such as DWI, Perfusion-weighted Imaging (PWI), and CT Perfusion (CTP) provide valuable information to assess tissue viability and predict stroke outcomes. DL models have emerged as powerful tools in this domain, leveraging the ability to extract complex patterns and relationships from high-dimensional imaging data. Studies in this area have mainly used U-Net-like CNN network architectures and incorporated different training strategies and modules such as multi-scale input [54, 71], ensemble learning [68, 81], transformer [84] and attention modules [87, 88].

[67] presented a deep learning model trained on randomly sampled local patches extracted from Tmax feature maps of MRI scans acquired immediately after symptom onset. Their approach outperformed traditional single-voxel regression models, such as [94], in predicting tissue survival outcomes in acute ischaemic stroke patients. Similarly, [69] developed a generalised linear model, a shallow CNN and a deep CNN based on the SegNet [95] model, trained on MRI-based perfusion maps of 222 patients. Their results demonstrated that the deep CNN model outperformed other models in predicting final infarct volumes.

The integration of various data sources shows great potential for improving the performance of DL models. [71] provided an example of this approach by proposing a method that used MRI maps and clinical data to predict the outcome of stroke lesions at a 3-month follow-up. The model achieved improved accuracy by incorporating clinical information at both the population and individual patient levels. [88] developed a novel DL model using cross-attention to incorporate the spatio-temporal nature of 4D CTP imaging and clinical variables to predict final infarct. Although their multimodal model performed slightly worse, with a Dice Similarity Coefficient (DSC) score of 0.25, compared to their unimodal model DSC score of 0.26, it significantly outperformed the unimodal approach in estimating lesion volume (mean error: 19 ml vs. 35 ml). In addition, the model generated attention maps and valuable information on how clinical variables influence stroke outcomes at individual patient level.

**Fig. 2:**
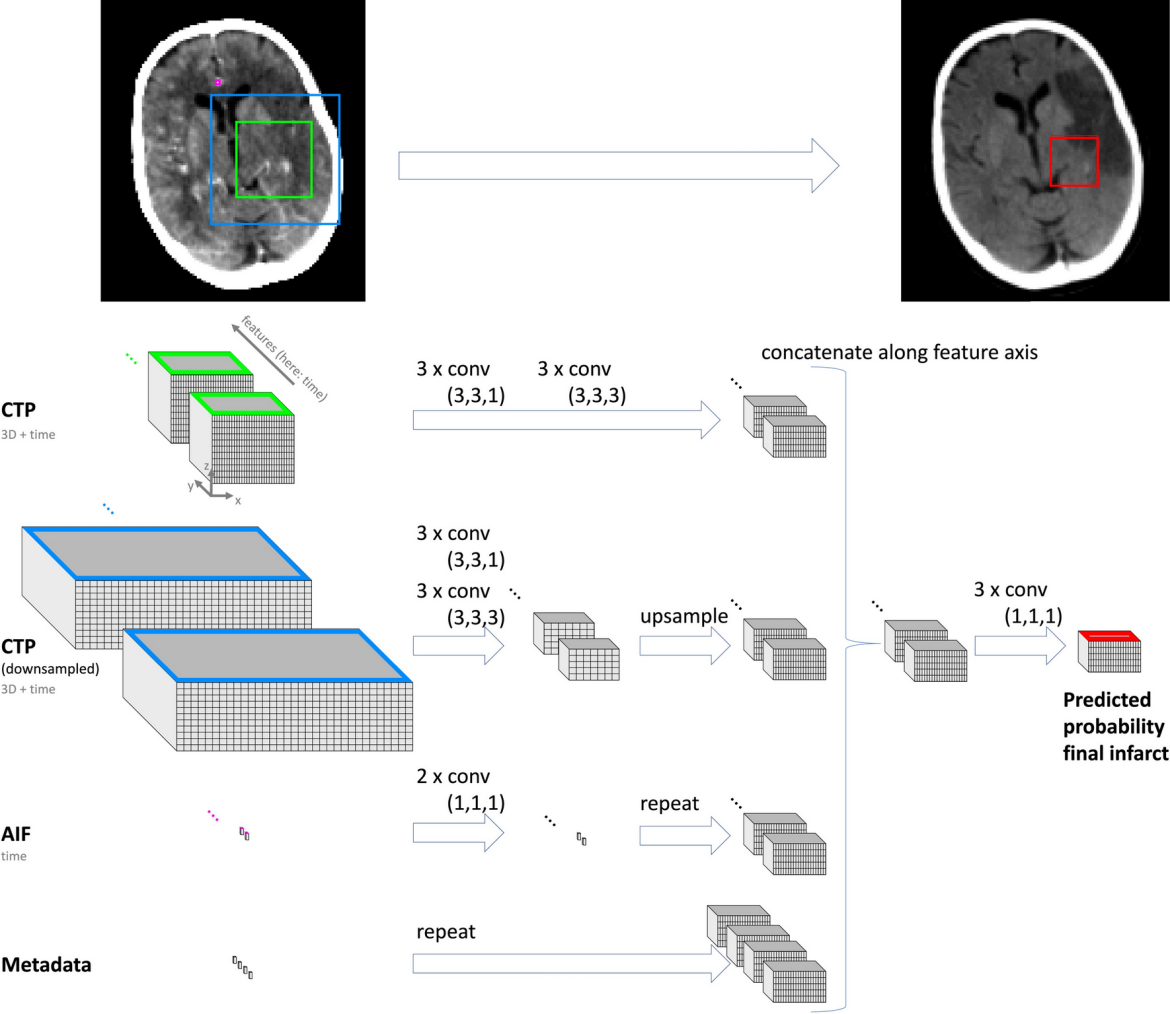
The multimodal architecture introduced in [78]. The network exploits imaging and clinical information to predict the follow-up stroke lesion. CTP: computed tomography perfusion, AIF: arterial input function which refers to the measure of blood concentration that enters the affected brain tissue after a stroke, Metadata: clinical information *e.g. * time to scanning, recanalization, completeness of recanalization. Yellow and blue rectangles represent patches extracted from CTP scan, purple rectangle represents AIF in the volume of interest and red rectangle is the target region of interest to segment lesion on follow-up NCCT scan. Figure from [78]

[78] developed a CNN model, based on DeepMedic [96], using spatio-temporal CTP data and clinical information to predict follow-up infarct lesions, as shown in Fig. 2. The model architecture comprises four branches that extract the features of CTP images and their downsampled versions, arterial input function, and metadata. These features are concatenated and fed through three convolutional layers to generate the final infarct segmentation map. [54] investigated CNNs to predict stroke infarct volume, incorporating reperfusion status (successful or failed blood flow restoration) into the model. Their findings suggest that CNNs outperform the current clinical standard, the perfusion-diffusion mismatch model [97], in predicting infarct volume for both reperfused and non-reperfused patients (reperfused patients: Area Under the Curve (AUC) = 0.87 vs. 0.79; non-reperfused patients: AUC = 0.81 vs. 0.73, in CNN vs. perfusion-diffusion mismatch models, respectively). [79] demonstrated the effectiveness of integrating information from perfusion dynamic susceptibility MRI (DSC-MRI) and parametric maps in predicting ischaemic stroke tissue outcomes. Their end-to-end architecture, which is based on U-Net and fully CNN, extracted features from raw perfusion DSC-MRI to complement standard parametric maps, achieving competitive results in ISLES 2017 competition [43] and reaching the second highest average DSC of 0.31.

To improve model performance, researchers have explored ensemble approaches that integrate several deep neural networks or different configurations within the same architecture. A key example was presented in [68], where an ensemble model of various configurations of a U-Net architecture, including different patch sizes (multi-scale image patches), number of patches, and number of convolutional filters with a multi-step training strategy was employed to overcome challenges of class imbalance due to lesion size compared to whole brain, and limited data of 30 training cases. This approach, utilised multi-scale image patches, and achieved outstanding performance in predicting both lesion outcome and clinical outcomes, ranking amongst the leaders in the ISLES 2016 Challenge [43]. Similarly, [81] introduced AUNet, an ensemble model that integrated DL and ML techniques. AUNet brought together an adaptive linear ensemble model (ALEM), incorporating RF, extremely randomised trees, and eXtreme Gradient Boosting (XGB), with a U-Net [98] to capture voxel-wise information and learn spatial relationships. Their method effectively predicted infarct volume in patients with acute ischaemic stroke under varying recanalization conditions, achieving an AUC of 0.898 on a dataset of 40 patients.

**Fig. 3:**
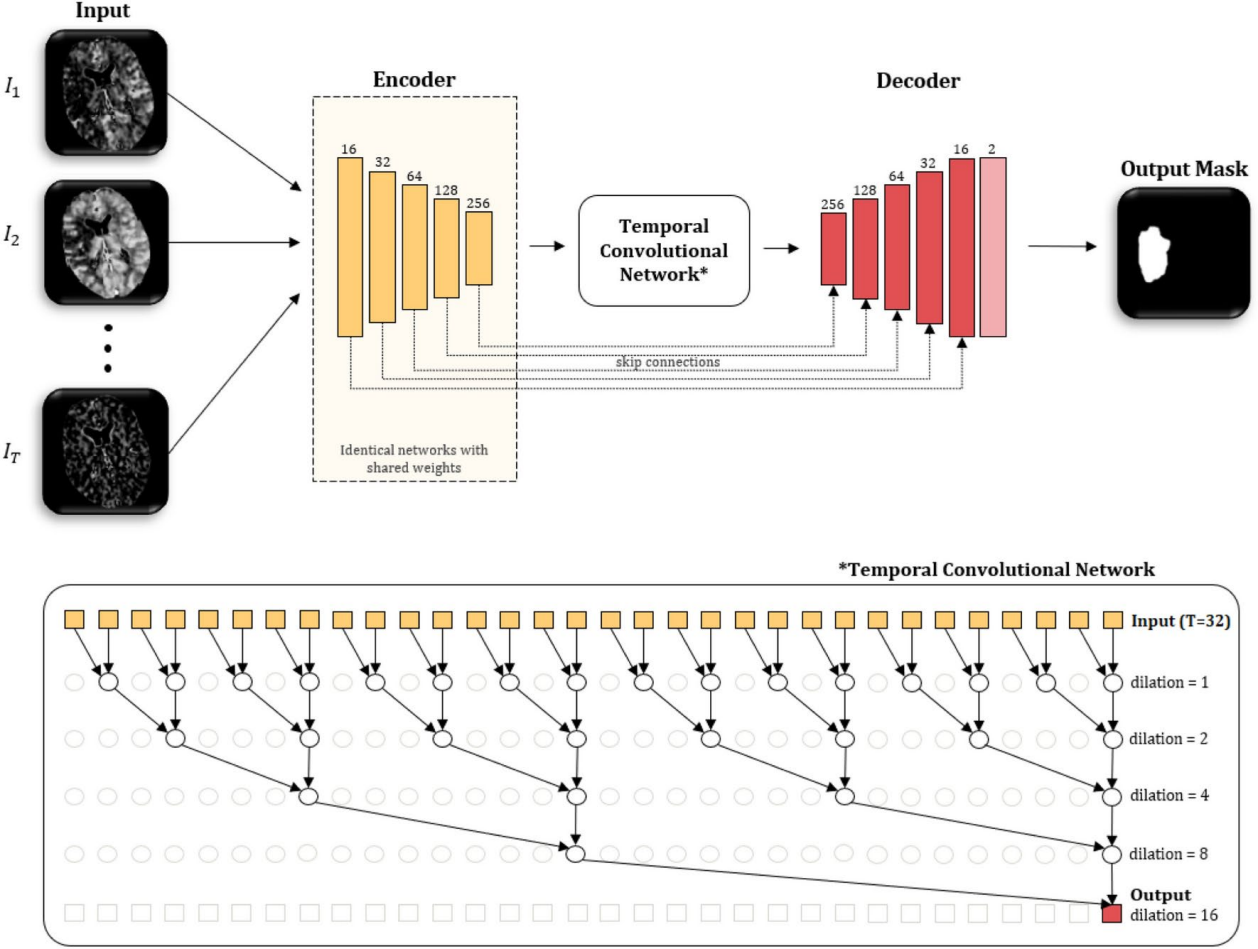
The overview of the temporal U-Net proposed by [29] to estimate the final lesion mask of stroke. Each CTP scan is fed separately into an encoder, and the temporal convolutional block encodes temporal information and passes the features to the decoder for the purpose of predicting the final lesion mask. Figure from [29]

Recent research shows that DL models can directly analyse raw 4D CTP data to predict stroke lesion outcomes, removing post-processing steps to generate 3D perfusion parameter maps and potentially capturing spatio-temporal information more effectively [29, 84]. [29] demonstrated the benefit of using spatio-temporal information of raw 4D CTP by employing a U-Net-like architecture (see Fig. 3) with shared-weight encoders for spatial feature extraction, followed by a temporal convolutional network (TCN) to integrate temporal information. A decoder then generated a segmentation map which was then post-processed for refinement. Their 3D+time model, validated through a 10-fold cross-validation on 147 patients curated from ERASER [50] and custom data, obtained a notably higher mean DSC of 0.45 compared to both their 2D+time version (0.43 DSC) and the state-of-the-art method [69] based on perfusion maps (0.38 DSC).

**Fig. 4:**
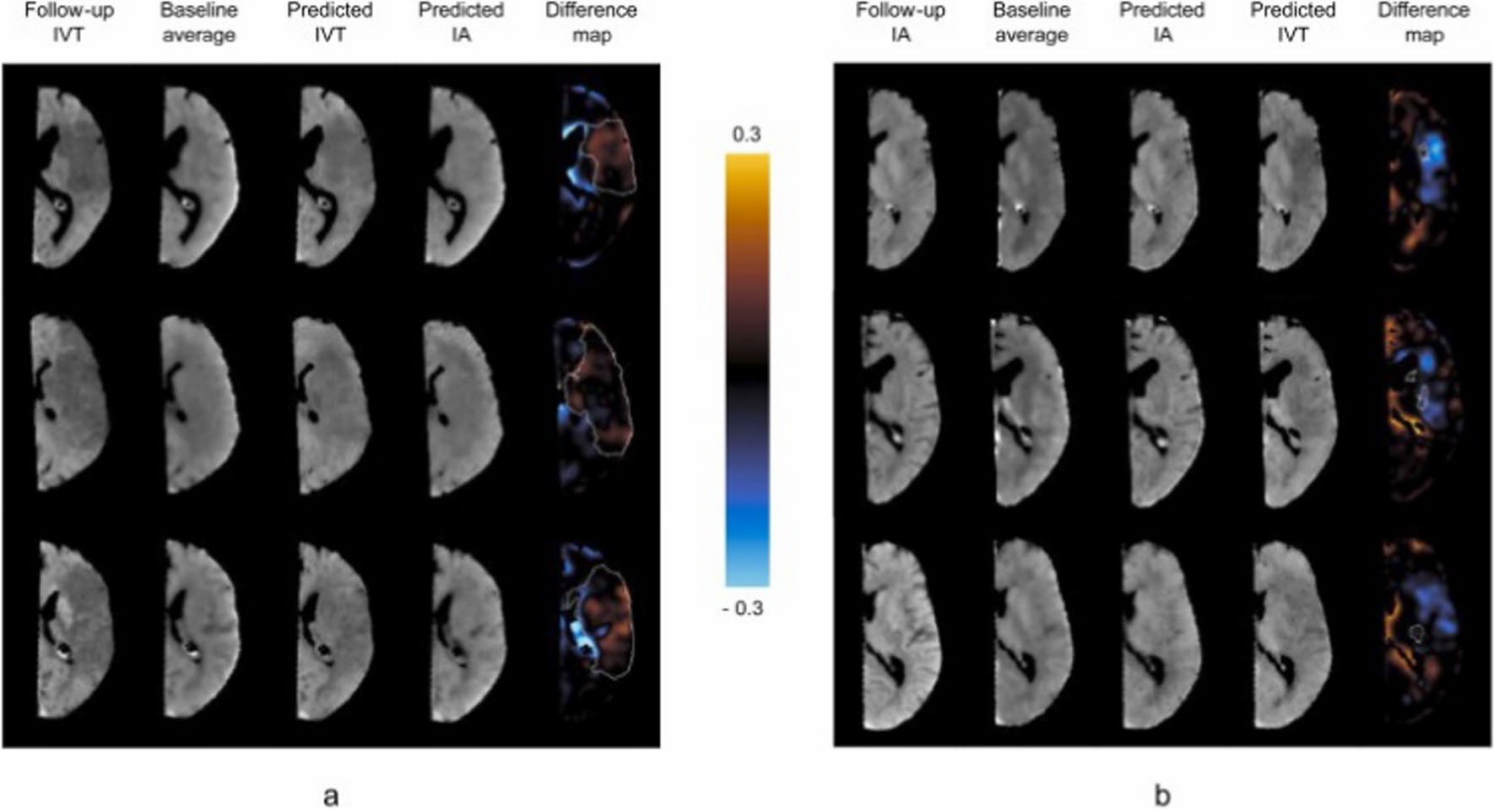
Sample results of [90] for three patients from each treatment group of **a** thrombolysis (IVT) and **b** thrombectomy (IA). Five columns for each patient: (1) the original follow-up CT image, (2) the baseline image average, (3) the CGAN prediction for the patient’s treatment group, (4) the CGAN prediction from the alternative treatment model, and (5) a difference map highlighting changes in intensity between predictions. The ground truth lesion mask is outlined in white within the difference map, with orange indicating increased intensity in the alternative treatment prediction (column 4) and blue indicating decreased intensity. Figure from [90]

[90] introduced an annotation-free method that directly predicted follow-up NCCT images of acute ischaemic stroke patients from 4D CTP scans, bypassing the need for complex perfusion analysis or manual annotation of segmentation maps. Their proposed method first employed a temporal autoencoder network to extract features from CTP, followed by a Conditional Generative Adversarial Network (CGAN) that utilised these features to directly predict follow-up NCCT images. The model was trained and tested on the same dataset of 147 patients as in [29], with separate models for thrombolysis (45 patients) and thrombectomy (102 patients) treatments. Their findings showed that their approach produced realistic follow-up image predictions (also see Fig. 4) with a performance comparable to the previous state-of-the-art method [29] which relied on manual annotation. Specifically, the 3D-TCN method in [29] achieved DSC scores of 0.396 and 0.214 for thrombolysis and thrombectomy, respectively, while [90]’s method achieved DSC scores of 0.375 and 0.214.

**Fig. 5:**
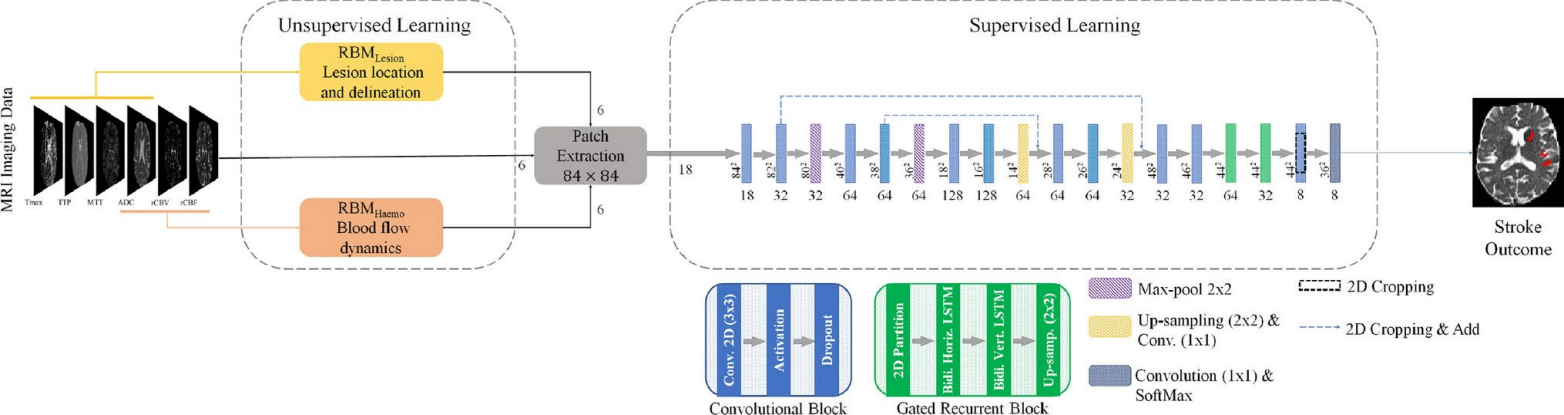
The architecture proposed by [27] to predict stroke outcome. The first part of the network includes unsupervised training that uses two Restricted Boltzmann Machines (RBM) and the second part of the network comprises of CNN and gated-Recurrent Neural Network (RNN)s for supervised training. Figure from [27]

Some recent studies explored different techniques to improve model performance. For example, [76] integrated an attention gate into a U-Net network [99] to predict tissue outcome from MRI perfusion maps and obtained AUC of 0.92 and DSC of 0.53 on a dataset consisting of iCAS [100] and DEFUSE-2 [46] data. [27] achieved the best performance with DSC of 0.38 in the ISLES 2017 [43] dataset by employing a two-stage approach (see Fig. 5), initially using Restricted Boltzmann Machiness (RBMs) in an unsupervised manner to identify lesion locations and blood flow characteristics, followed by a supervised stage where a CNN based on U-Net [98] and gated-RNNs were employed to extract spatial relationships and predict stroke lesions on follow-up scans.

Synthetic data has also emerged as a valuable tool for training models, especially when real-world data is scarce. Researchers have shown the potential of synthetic data in this context by using physically realistic simulated perfusion MRI data to train models for predicting final infarct lesions [73]. Furthermore, incorporating patient-specific details, such as arterial input functions, has demonstrably improved the accuracy of these predictions [75]. Generating synthesised data effectively addresses data limitations to potentially better integrate domain knowledge into DL models.

### Functional outcome (mRS) prediction

The studies in this category aim to assess the functional abilities of stroke patients, following intervention, by analysing unimodal and multimodal data to estimate their mRS scores. These learn to establish the stroke treatment outcome as *dichotomous* (mRS scores 0-2 as favourable and mRS scores 3-6 as unfavourable) or as *individual* mRS scores (0-6). At the inference stage, the model predicts the functional outcome for a new patient using imaging and/or clinical data. Consequently, these models can help clinicians determine the most appropriate treatment.

#### Image data analysis

Predicting the functional outcome after stroke treatment is essential for guiding treatment decisions and patient management. Neuroimaging biomarkers and clinical information have commonly been used, but recent advances in DL have introduced new opportunities to use imaging data. When clinical health records are not accessible during hospital admission, the use of imaging data offers a viable alternative to evaluate stroke outcomes.

Table 5 shows the studies that leverage only imaging information to predict the functional outcome of stroke treatment. These studies employ various DL techniques, including CNN [33, 101], Siamese networks [102], and attention mechanisms [103]. The models are trained on CTA [26], NCCT [101, 104], MRI [105] (including multi-parametric MRI [102]), and diffusion-weighted imaging [33, 106] modalities.

**Fig. 6:**
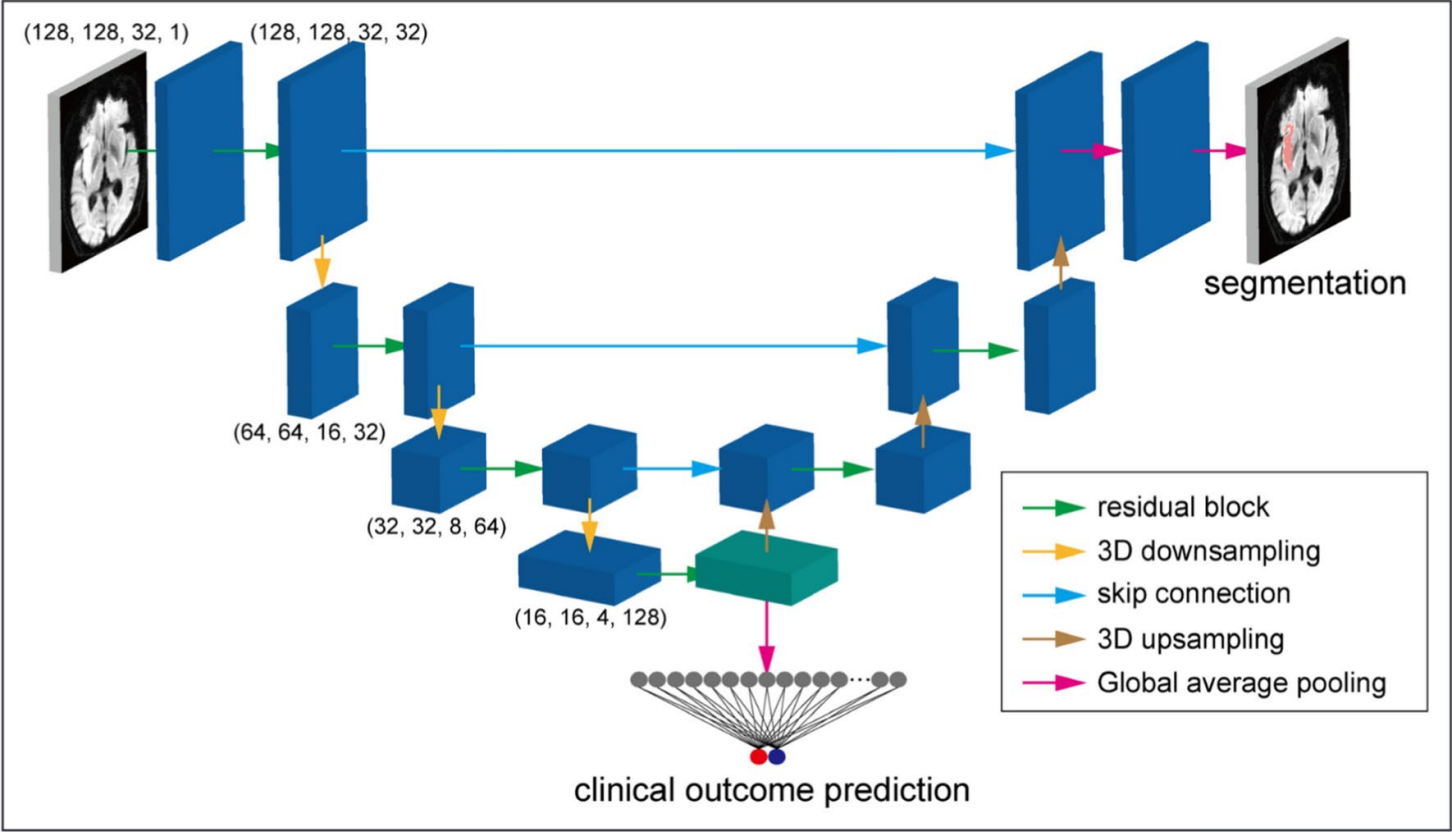
A multi-task model introduced by [33] for lesion segmentation and clinical outcome prediction. In the bottleneck of the U-Net model, binarised mRS scores are predicted and the output of the decoder is the lesion core (indicated in red). Figure from [33]

[26] used a ResNet model with structured Receptive Field Networks (RFNNs) [107] which was trained on 2D images, to extract high-level features from a projection of the maximum intensity of a 3D CTA volume of the patient. Their model outperformed traditional biomarkers such as ASPECTS and ischaemic core volume to predict reperfusion (TICI) (AUC 0.65) and functional outcome (AUC 0.71) on the MR CLEAN registry [52] dataset. In addition, they used Gradient-weighted Class Activation Map (Grad-CAM) [108] to interpret their model result. Grad-CAM demonstrates the convolutional feature maps that contribute the most to prediction in input space. [33] combined lesion segmentation and mRS score prediction tasks in a single U-Net model trained on DWI as seen in Fig. 6. Using the features at the U-Net bottleneck, they performed the classification task of mRS scores, and the output map of the U-Net decoder was used for the segmentation of the infarct core. Their model predicted functional outcome at 90 days, outperforming standard biomarkers like the ASPECTS and ischaemic core volume by achieving an AUC of 0.81 in a derivation cohort of 250 patients and AUC of 0.73 in a validation cohort of 74 patients. Their dataset was collected from four hospitals in Japan and is available on request from the authors.

**Table 5:** Overview of the studies that use only imaging information to predict functional outcome (mRS scores) as stroke treatment outcome.

Study	Year	Patient subgroup	Treatment type	Method	Input modality	Number of patients	Data split	Best result
[26]	2019	AIS	EVT	ResNet	CTA	1301	4-fold	AUC: 0.71
				+ RFNN			cv	
[33]	2020	LVO	EVT	U-Net	DWI	250	5-fold cv	AUC: 0.81
				+ FC			74 ext. val.	
[102]	2020	AIS	EVT	Siamese	mpMRI	43	70:30	ACC: 0.67
				Network				
[105]	2022	N/R	N/R	CNN	MRI	44	90:10	ACC: 0.932
				VGG16				
[104]	2022	Posterior	EVT	ResNet-18	NCCT	31	5-fold	AUC: 0.74
		Circulation	rtPA	SegNe			cv	
[106]	2022	AIS	EVT	CAE+	DWI	206	41 test	AUC: 0.88
			tPA	SVM			cases	
[103]	2022	LVO	EVT	CNN +	DWI	322	LOOCV	AUC: 0.83
			tPA	Attention				
[101]	2023	sICH	N/R	CNN	NCCT	377	91 ext. val	ACC: 0.706
[109]	2023	AIS	N/R	ResNet	CT	3573	80:20	ACC: 0.788
				DDPM			5-fold cv	
[110]	2024	AIS	EVT	ResNet50	DWI	3338	60:20:20	AUC: 0.788
			tPA		ADC			
[111]	2024	AIS	N/R	CNN,LSTM	DSC-PWI	88	10-fold cv	Mean Score:
				RNN				0.971

Acute Ischaemic Stroke (AIS), Large Vessel Occlusion (LVO), Endovascular Therapy (EVT), Symptomatic Intracerebral Haemorrhage (sICH), tissue Plasminogen Activator (tPA), recombinant tissuetype Plasminogen Activator (rtPA), Convolutional Neural Network (CNN), Convolutional Auto Encoder (CAE), Support Vector Machine (SVM), Denoising Diffusion Probabilistic Model (DDPM), CT Angiography (CTA), Diffusion-weighted MRI (DWI), Multiparametric MRI (mpMRI), Magnetic Resonance Imaging (MRI), Non-contrast Computed Tomography (NCCT), Computed Tomography (CT),Dynamic Susceptibility Contrast PWI (DSC-PWI), Leave-One-Out Cross-Validation (LOOCV), Cross-Validation (cv), Area Under the Curve (AUC), Accuracy (ACC), Not Reported (N/R)

[102] proposed a parallel multi-parametric feature embedded Siamese network (PMFE-SN) and achieved high accuracy even with a small number of samples (an accuracy of 0.67 with just two minority class samples for training data of the ISLES 2017 dataset [43]) and effectively handled class imbalance in their MRI data. [105] utilised a VGG-16 network trained on an MRI dataset of 44 patients, achieving an accuracy of 0.932 and 0.927 in the prediction of mRS and NIHSS respectively. Their dataset was collected from two hospitals in China and also available by request from the authors. [103] demonstrated that a CNN with an attention mechanism, trained on 322 DWI scans of one day post-stroke patients, gathered from ASTER [112] and INSULINFARCT [113] trials and the Pitié-Salpêtrière registry [114], achieved better performance (AUC of 0.83) compared to Logistic Regression (LR) models using lesion volume (AUC of 0.78) or ASPECTS (AUC of 0.77) in predicting long-term functional outcomes at three months post-stroke.

Other researchers have explored DL to extract more nuanced information from imaging data. [106] investigated whether information beyond infarct volume from follow-up DWI could improve the prediction of the outcome in patients with acute ischaemic stroke. They found that classifiers using features extracted by a Convolutional Auto Encoder (CAE) or radiomics from follow-up DWI scans (extracted from HERMES [47], ISLES 2015 [115], and MR CLEAN-NO IV [53] datasets), achieved higher AUC values (0.88 and 0.81, respectively) compared to a model based solely on infarct volume (AUC of 0.79). [109] introduced a new approach that utilised a Denoising Diffusion Probabilistic Model (DDPM) to capture the evolution of stroke for predicting mRS and NIHSS at discharge from CT images in a dataset^2^ of 3573 AIS patients, based solely on initial patient scans. They also incorporated both longitudinal data and time since stroke onset and achieved AUC of 0.788 in mRS and AUC of 0.669 in next-day stroke severity (NIHSS).

#### Multimodal data analysis

Studies in the previous section have demonstrated the potential of using imaging data alone to predict stroke treatment outcomes. However, incorporating additional data from other modalities, such as neuroimaging data, imaging biomarkers and clinical information, has been shown to improve the accuracy of outcome prediction [116–118]. These multimodal (fusion) models capture complementary information from various data sources, that can potentially leading to better performance [93, 119, 120].

The outcome prediction studies using multimodal data are listed in Table 6. These studies employ various DL architectures, ranging from CNN, Long Short-Term Memory (LSTM) to Transformers. The models utilise diverse data sources, including imaging information (*e.g. * NCCT, CTA, CTP) and clinical variables (*e.g. * demographics of the patients such as age and sex, and medical records detailing specifying like diabetes, hypertension and medications, etc.).

**Table 6:** Overview of the studies that perform functional outcome as stroke treatment outcome and use imaging and clinical information.

Study	Year	Patient subgroup	Treatment type	Method	Image modality	Clinical features	Number of patients	Data split	Best result
[68]	2016	AIS	N/R	U-Net	MRI	3	49	19 test	AUC: 0.74
				+ FC	Sequences			cases	
[119]	2020	AIS	tPA	CNN+	NCCT	N/S	204	85:15	AUC: 0.75
				ANN					
[116]*	2020	IS	EVT	CNN+	NCCT	27	500	80:20	AUC: 0.75
				Attention					
[121]	2020	AIS	N/R	CNN+	Textual	MRI	1840	70:30	AUC: 0.75
				NLP	Features	Reports			
[120]*	2020	Acute	N/R	CNN+	MRA	7	316	63 test	AUC: 0.76
		cerebrovascular		MLP				cases	
[117]	2021	Acute	Reperfusion	CNN	DWI	N/S	1438	75:25	AUC: 0.975
		Brainstem	Therapy		ADC				
[35]*	2022	AIS	EVT	CNN	NCCT	27	500	75:25	AUC: 0.82
[122]*	2022	AIS	EVT	CNN	CTA	50	3279	5-fold	AUC: 0.81
				(ResNet10)				cv	
[123]	2022	Proximal	EVT	CNN+	MRI	4	119	5-fold	AUC: 0.77
		occlusion		LSTM	Sequences			cv	
[93]	2023	AIS	EVT	CNN	DWI	13	640	70:15:15	AUC: 0.92
			tPA					280 ext. val	
[118]*	2023	MCA	EVT	CNN+	DWI,	32	222	50 test	AUC: 0.766
				ANN	PWI			cases	
[124]*	2023	MCA	tPA	CNN+	CTP	10	230	80:20	AUC: 0.865
								patients	
[125]	2023	ICH	N/R	NLP	EHR	N/S	1363	75:25	AUC: 0.914
				DL, ML					
[126]	2023	AIS	EVT	CNN	CTA	N/S	44	N/S	AUC: 0.874
[30]*	2023	AIS	EVT	Transformer,	NCCT	27	500	75:25	AUC: 0.85
				CNN					
[127]	2023	IS	EVT	CNN	NCCT,CTA	6	460	4-fold	AUC: 0.807
				L2GAN	CTP			cv	
[128]*	2023	IS	EVT	CNN	NCCT	8	743	10-folds	AUC: 0.806
			tPA	Siamese				cv	
[129]	2023	AIS	N/R	ResNet,	CTP	14	98	60:20:20	AUC: 0.75
				DAFT [130]					
[131]	2023	AIS	N/R	DenseNet	DWI	3	4147	80:20	AUC: 0.786
					ADC			5-fold cv	
[132]	2024	AIS	N/R	ResNeXt	DWI	22	2606	5-fold	AUC: 0.83
				+ CBAM	FLAIR			cv	
[133]*	2024	AIS	EVT	DenseNet121	NCCT	26	975	80:20	AUC: 0.811
				LR					
[134]	2024	AIS	N/R	DL	DWI	N/S	80	N/S	ACC: 0.36
[135]*	2024	AIS	EVT	GCN	CTA	81	220	5-fold	AUC: 0.87
				FCN	CTP			cv	
[136]	2024	AIS	EVT	ResNet	NCCT	7	1335	6-fold	AUC: 0.91
								cv	
[137]*	2024	AIS	EVT	CNN	CTP	9	70	10-fold	AUC: 0.77
				Cross-Attention				cv	

Ischaemic Stroke (IS), Acute Ischaemic Stroke (AIS), Middle Cerebral Artery (MCA), Intracerebral Haemorrhage (ICH), Endovascular Therapy (EVT), tissue Plasminogen Activator (tPA), Deep Learning (DL), Machine Learning (ML), Convolutional Neural Network (CNN), Artificial Neural Network (ANN), Multi Layer Perceptron (MLP), Natural Language Processing (NLP), Long Short-Term Memory (LSTM), Magnetic Resonance Imaging (MRI), CT Perfusion (CTP), Diffusionweighted MRI (DWI), Perfusion-weighted Imaging (PWI), Non-contrast Computed Tomography (NCCT), CT Angiography (CTA), Apparent Diffusion Coefficient (ADC), Electronic Health Records (EHR), Cross-Validation (cv), Area Under the Curve (AUC), Not Reported (N/R), Not Specified (N/S). * indicates that the code used in the study is available

[68] proposed an ensemble of deep neural networks that achieved AUC of 0.74 on ISLES 2016 [43] dataset in clinical outcomes after ischaemic stroke treatment. [116] used a multimodal CNN approach, incorporating an attention mechanism to capture inter-dependencies among features, to predict the success of endovascular treatment for ischaemic stroke patients from NCCT acquired at the hospital admission. Their model achieved AUC of 0.75 in dichotomised mRS scores and accuracy of 0.35 in individual mRS scores prediction on MR CLEAN dataset. [93] developed a fusion model that combined DWI and clinical features extracted using a ResNet and SVM model respectively, to predict 90-day functional outcomes. Their model achieved 0.92 AUC on a dataset of 640 patients that included four multi-centre trials (iCAS, DEFUSE-2, DEFUSE-3, CRISP) and two single centre registries (University of California, Los Angeles stroke registry and Lausanne University Hospital stroke registry), demonstrating better performance than models based imaging features (AUC of 0.88) or solely on clinical (AUC of 0.88). [124] used a CNN which included five convolutional layers along with an efficient channel attention (ECA) layer [138] to encode features of CTP maps and combined them with demographic data features to improve post-thrombolysis functional outcome prediction. Their model was trained on 230 patients and validated on 129 patients, and achieved an AUC of 0.865 in a multimodal configuration, surpassing the performance of models that relied only on imaging data (AUC of 0.792) and clinical data (AUC of 0.670). No information is provided on the availability of their dataset.

In [34], Bacchi et al. used a dataset consisting of 204 patients with NCCT image volumes and relevant clinical information, such as age, gender, blood pressure, and NIHSS scores, to predict the binary outcomes of thrombolysis treatment with both mRS at 90 days and NIHSS at 24 h. They found that their most successful model was a combination of a custom CNN comprising two 3D convolution layers followed by a maximum pooling layer and two Fully Connected (FC) layers and an Artificial Neural Network (ANN) based on 3 FC layers and ReLU activation function that were trained on both NCCT image volumes and clinical metadata. This model reached an AUC of 0.75 in mRS and 0.70 in NIHSS score prediction. [34] also did not make their data available.

[122] investigated the use of ML and DL to predict clinical outcomes in stroke patients undergoing EVT. They used both imaging and clinical data from the MR CLEAN registry and explored two approaches: one using radiomics features extracted from specific brain regions and the other employing 3D DL models analysing whole brain images combined with clinical information by using ML (RF, SVM, XGB, ANN) and DL (ResNet10) respectively. Interestingly, contrary to other studies, incorporating imaging data did not significantly improve the performance of mRS prediction. Using only clinical data, AUC was 0.81 for ML and 0.77 for DL, while when using both imaging and clinical data, the AUC remained at 0.80 for ML and 0.77 for DL. However, for TICI score prediction, the inclusion of imaging data increased the AUC from 0.53 to 0.57 for ML and from 0.53 to 0.61 for DL.

**Fig. 7:**
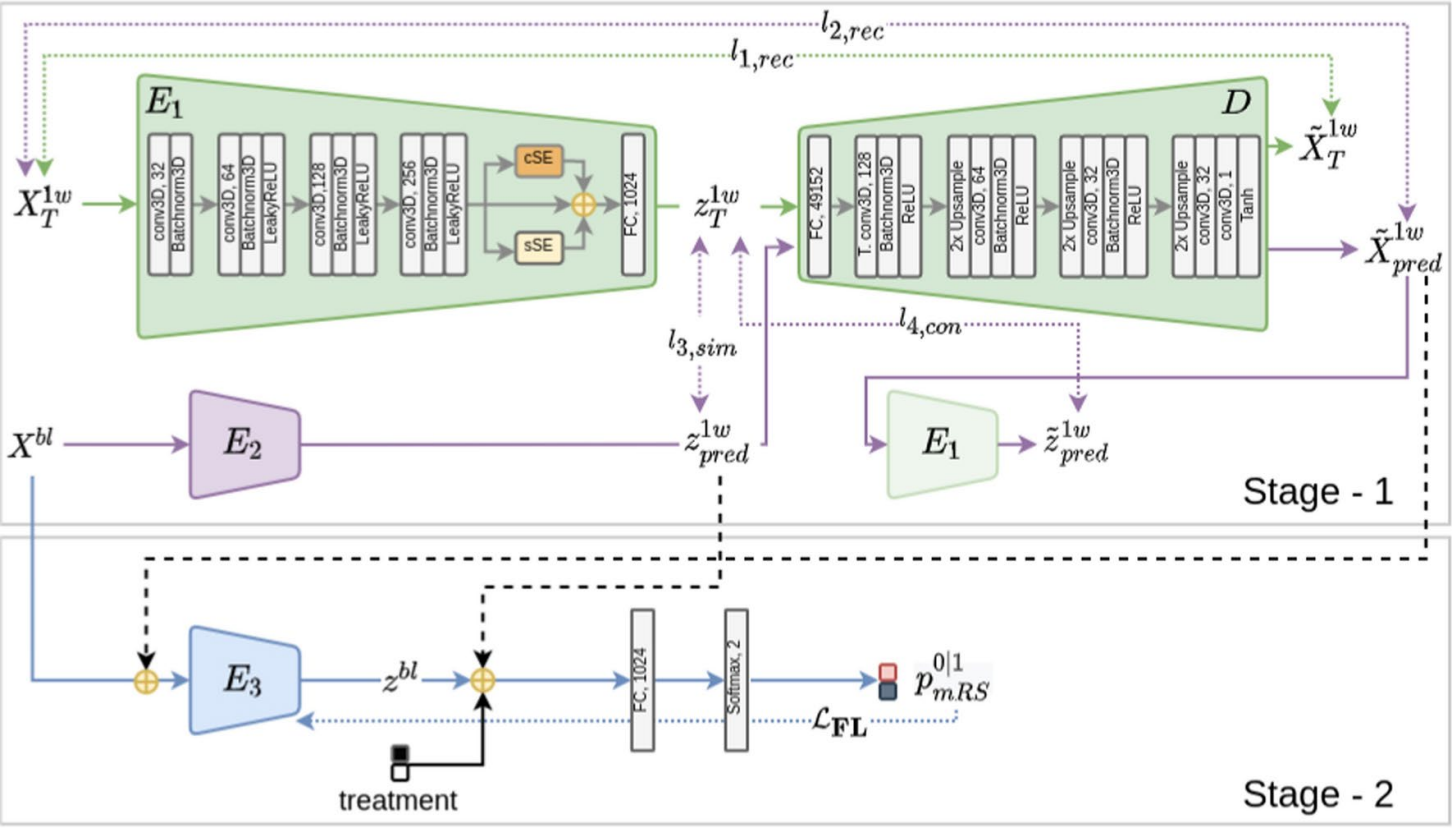
Overview of the FeMA network [35]. The model predicts the follow-up NCCT scan (1-week follow-up $X^{1w}_{T}$) from the baseline scan $X^{bl}$. Then, it combines the baseline scan with the predicted follow-up scan $X^{1w}_{pred}$ and 1-week follow-up scan features $z^{1w}_{pred}$ to predict the functional outcome of the stroke treatment. Figure from [35]

Early prediction (at one week) of stroke evolution has been found to be critical for predicting the functional outcome after stroke treatment. [36] highlighted this by demonstrating a correlation between the volume of lesions in 1-week follow-up NCCT scans with functional outcome. Building on this, [35] developed a model to predict mRS scores by incorporating follow-up scan data. Their approach, named Feature Matching Auto-encoder (FeMA) (see Fig 7), leveraged a self-supervised voxel-wise method based on a custom CNN model that consisted of three encoder and a decoder modules to predict follow-up scans in stroke patients from their baseline scan alone. In training their model, a two-step strategy was used where in the first stage a 1-week follow-up scan was reconstructed from a baseline scan to encode information about tissue changes over a week after treatment, and then in the second stage, the learnt features from the first stage and a baseline scan were combined to estimate mRS scores. The model was tested using the MR CLEAN trial dataset and obtained an AUC of 0.82.

Interpretable deep learning models have also been developed to improve the estimation of functional outcomes. [118] used an interpretable DL model based on the study of [139] and demonstrated that utilising both imaging data (DWI) and clinical variables (*e.g. * age, systolic blood pressure, diabetes, hypertension, smoking). They collected a dataset of 222 patients from Inselspital Bern, Switzerland which is available by requests from the authors. Their model achieved an accuracy of 0.72, outperforming models based only on imaging data (accuracy of 0.614) or clinical data (accuracy of 0.60). The inclusion of imaging data allowed the model to significantly outperform experienced stroke neurologists, who had an accuracy of 0.64 with imaging and clinical data, an accuracy of 0.55 with imaging data only and an accuracy of 0.60 with clinical data alone.

A recent study by [135] introduced an uncertainty-aware graph deep learning model to predict long-term functional outcomes and mortality rates in AIS patients following EVT. Their dataset was collected from the University Hospital of Erlangen, comprising 220 AIS patients, and is available by request from the authors. They employed Graph Convolutional Networks (GCNs) and Fully Connected Networks (FCNs) to predict the outcome at admission, post-EVT, and 24 h after stroke. Their results demonstrated comparable performance across the algorithms including LR, RF, XGB and FCN, with a maximum AUC of 0.87 for the prediction of mRS using GCNs. The performance of the predictions increased when data from later time points were incorporated, rising from an AUC of 0.76 at admission to 0.84 post-EVT and to 0.87 at 24 h post-stroke.

In addition to using imaging and clinical data, Natural Language Processing (NLP) has been used in several medical domains to analyse clinical narratives [140] and applied to treatment outcome prediction. By generating text-based markers from free-text MRI reports, NLP-based ML algorithms have demonstrated improved performance in predicting poor outcomes in AIS patients [121]. Similarly, NLP techniques have been applied to enhance the prediction of functional outcomes after Intracerebral Haemorrhage (ICH) [125].

## Discussion

So far in this article, we have reviewed the current landscape of stroke outcome prediction methods that have explored deep learning to make estimates at various time-points following a stroke. Next, we present our key observations with respect to the major issues and challenges, and discuss current limitations.

### Use of deep learning

A consistent feature across the reviewed studies is the dominance of using DL models based on CNNs, for example [29, 35, 69, 78, 89, 102, 109, 133], with U-Net variations commonly used for final infarct prediction [29, 54, 68, 70, 83, 89], due to their ability to handle segmentation tasks effectively, whereas ResNet-style architectures are preferred for predicting functional outcomes, such as [26, 104, 110, 122, 129]. Among imaging modalities, MRI is most used for both the prediction of the final infarct and functional outcome, as it provides detailed information about stroke tissue, *e.g. * [27, 67, 69, 87]. CTP is popular for final infarct prediction due to its ability to assess cerebral blood flow, a key factor in tissue infarction [70, 78, 83, 88]. NCCT is also frequently used for predicting functional outcomes, likely due to its greater availability and lower cost, *e.g. * [30, 34, 101, 116, 133].

A common trend in the reviewed studies is the development of novel implementations rather than investigation and validation of existing methods [141]. Furthermore, many studies lack sufficient detail regarding model building procedures, data processing steps and hyperparameter tuning. In addition to this problem, only a small proportion of studies (15 out of 60) analysed in this research share their code publicly, such as [27, 84, 116, 124, 133] as can be seen in Table 7. Consequently, ensuring transparency and reproducibility remains a significant challenge. To address this, a commitment to open science practices is necessary.

Finally, while DL models have shown promising performance in predicting stroke outcomes, their lack of interpretability makes it difficult to assess their reliability and adoption in clinical settings [141]. Clinicians expect to better understand "black-box" DL models, so they can have confidence in and rely on their predictions [90, 118]. To address this critical gap, a few recent studies [90, 103, 118, 135] have explored interpretable DL models in the context of SOP.

### Multimodal data

The majority of studies, particularly the studies on functional outcome prediction, leverage multimodal data, combining imaging and clinical information to achieve superior performance compared to models that rely solely on unimodal information [30, 34, 93, 116, 118]. This approach acknowledges that the prediction of stroke outcomes depends not only on the extent of brain damage, but also on previous health conditions and other characteristics of the patient. Furthermore, incorporating follow-up (lesion outcome) information demonstrably improves the performance of models to predict mRS scores, such as [35, 106].

### Availability of data & benchmarks

Although DL techniques such as CNNs, and more recently transformer-based architectures, hold significant promise for predicting stroke outcomes, several limitations need to be addressed. A significant challenge is the limited availability of large, all-encompassing, easily accessible and well-annotated datasets [8, 141] that can allow DL algorithms to learn complex patterns and ensure generalisability. The majority of the studies reviewed in this research use data sets consisting of fewer than 500 patients. This scarcity restricts the development of robust and generalisable models.

While significant efforts have been made to develop clinical trial datasets such as MR CLEAN [51], HERMES [47], ERASER [50] and DIFUSE-2 [46], are not structured as benchmarks for comparative evaluation of DL models. This lack of standardised benchmarking framework (datawise and processes) has led to various niche datasets, inconsistent evaluation metrics and experimental setups, hence making it challenging to assess relative strengths and weaknesses between different algorithms fairly and objectively. Even when employing the same clinical trial dataset, inconsistencies arise due to the incomparable use of data modalities and subsets for training and testing *e.g. * [26, 122]. Clearly, collaborative efforts are needed to establish comprehensive standardised public datasets and evaluation protocols that allow researchers to perform thorough comparisons and accelerate progress in model development.

Although some clinical trial datasets offer data from multiple centres, many single-centre studies rely on in-house datasets, such as [80, 82, 104, 111, 142], which often lack heterogeneity. Stroke presentation, imaging equipment and neuroimaging parameters vary considerably across healthcare institutions, and this in-house approach fails to capture this essential diversity. Such lack of diversity in single-centre training data limits the generalisability of models to real-world scenarios with broader patient characteristics and clinical practices. It also leads to limited or no means of external validation which creates a significant obstacle to clinical translation [141]. Such lack of external validation, which has been explored by only a few [33, 85, 101, 124], is cause for concern on the reliability of reported results.

## Future directions

There is scope for several promising areas of research to significantly improve prediction accuracy and robustness for stroke outcome using deep learning.

### Adaptive multimodal data fusion

Combining information from various data sources, consistently improves the predictive performance of models [34, 35, 132]. However, current methods often rely on naive fusion techniques, such as late fusion, which fail to explore the the complex relationships between different types of data [143, 144]. This suggests a critical need for the development of adaptive multimodal fusion methods that dynamically learn relationships and contextually integrate information, such as neuroimaging data and clinical records.

### Leveraging final infarct information

The information available in brain scans within post-treatment stroke lesions (final infarct) - specifically the size and location of the damaged area - is extremely valuable for predicting how well a patient will recover in the long term. While encoding final infarct information into models has shown promise for improving functional outcome prediction in some studies [35, 106], this area still requires further investigation. Therefore, developing models that can effectively encode such information, along with longitudinal lesion changes, could enhance prediction robustness.

### Federated learning

Federated learning presents a promising solution to the challenge of data heterogeneity in developing robust models for SOP. This decentralised approach allows for collaborative model training across multiple institutions without compromising patient privacy by avoiding sharing source data [145]. By enabling the training of models on larger and more diverse datasets, federated learning facilitates the development of more generalisable models capable of handling the variability encountered in real-world clinical settings [146]. Such collaborative approach holds significant potential for improving the performance and reliability of models in SOPs across diverse patient populations.

### Annotation-free segmentation

Currently, the reliance on manual lesion segmentation to generate ground truth labels in follow-up scans presents a major bottleneck due to its time-consuming and labour-intensive nature. The development of annotation-free models for final infarct segmentation offers a significant opportunity to improve efficiency and cost-effectiveness [90]. This would also allow researchers to access larger training datasets, leading to the development of more robust and accurate predictive models.

## Conclusions

This paper has provided a review of deep learning approaches for stroke outcome prediction, encompassing both final infarct and functional outcomes, using imaging and multimodal data. We have presented the most recent techniques, datasets, evaluations, and code. While significant progress has been made, particularly in utilising multimodal data and advanced model architectures, specifically U-Net variations for lesion outcome segmentation and ResNet architectures for functional outcome prediction, several critical challenges remain. The scarcity of large, standardised benchmark datasets and inherent data heterogeneity restrict the development of robust and generalisable models. Furthermore, the limited use of external validation, inconsistencies in reporting practices, and the lack of interpretable models raise concerns regarding the clinical translation and reliability of existing approaches.

Despite these challenges, several promising avenues for future research are: the development of adaptive multimodal fusion methods that effectively leverage the complementary nature of diverse data sources, the incorporation of longitudinal lesion features, the adoption of federated learning techniques for improved data utilisation, and the exploration of annotation-free lesion labelling methods.

## Appendix: Studies with code

See Table 7.

**Table 7:** The list of the studies that have shared their code publicly

Study	Task	Code link
[70]	Final Infarct	https://github.com/multimodallearning/stroke-prediction
[116]	Functional Outcome	https://github.com/zeynelsamak/Thrombectomy-Outcome
[120]	Functional Outcome	https://github.com/prediction2020/multimodal-classification
[27]	Final Infarct	https://github.com/apint0/stroke_prediction
[35]	Functional Outcome	https://github.com/zeynelsamak/FeMA
[122]	Functional Outcome	https://github.com/L-Ramos/mrclean_combination
[84]	Final Infarct	https://github.com/kimberly-amador/Spatiotemporal-CNN-Transformer
[86]	Final Infarct	https://github.com/sdlee087/deep_medical
[118]	Functional Outcome	https://github.com/liherz/functional_outcome_prediction_dl_vs_neurologists
[124]	Functional Outcome	https://github.com/Yutong441/deepCTP
[30]	Functional Outcome	https://github.com/zeynelsamak/TranSOP
[128]	Functional Outcome	https://github.com/GravO8/mrs-dl
[133]	Functional Outcome	https://github.com/jdddog/deep-mt
[135]	Functional Outcome	https://gitlab.cs.fau.de/gu47bole/ais
[137]	Functional Outcome	https://github.com/kimberly-amador/Multimodal-mRS90-Outcome-Prediction

## Footnotes

1 International Conference on Medical Image Computing and Computer Assisted Intervention - https://miccai.org/.

2 https://www.hra.nhs.uk/planning-and-improving-research/application-summaries/research-summaries/prediction-of-stroke-outcome-using-brain-imaging-machine-learning/.
